# Supplementary material for: “Grumpy” or “furious”? arousal of emotion labels influences judgments of facial expressions
Source: PLoS One. 2020 Jul 1;15(7):e0235390. doi: 10.1371/journal.pone.0235390 (PMC7329125; doi:10.1371/journal.pone.0235390)
Supplement: S1 Appendix — (DOCX) [file pone.0235390.s001.docx]

**Appendix A: Pilot data**

All pilot data, including within-subjects *t*-test comparisons between low, moderate (“mid”) and high label arousal ratings.

|  |  | **HAPPY** |  |  | **SAD** |  |  | **ANGRY** |  |  | **SCARED** |  |
| --- | --- | --- | --- | --- | --- | --- | --- | --- | --- | --- | --- | --- |
| **Participant** | **Contented** | **Pleased** | **Elated** | **Down** | **Miserable** | **Distraught** | **Grumpy** | **Irritated** | **Furious** | **Worried** | **Afraid** | **Terrified** |
| 1 | 2.4 | 1.7 | 6.2 | 2.3 | 3.5 | 7 | 3.2 | 2.5 | 6.9 | 5 | 5.4 | 7 |
| 2 | 2 | 3 | 6 | 2 | 2 | 5 | 3 | 4 | 6 | 5 | 6 | 6 |
| 3 | 4 | 5.1 | 5 | 1.1 | 1 | 1.9 | 3 | 5.1 | 6.1 | 2 | 2 | 1.9 |
| 4 | 2.3 | 4 | 5.7 |  | 3.8 | 5 |  |  | 5 | 3.5 | 4 | 5.2 |
| 5 | 2.7 | 4 | 2.6 | 3.1 | 2.7 | 3.3 | 5.1 | 4.5 | 5.1 | 2.9 | 5.9 | 5.3 |
| 6 | 4 | 4.6 | 5.8 | 2.4 | 3.7 | 6.6 | 3.5 | 4.5 | 6.7 | 5.1 | 4.5 | 6 |
| 7 | 3.9 | 4 | 6.3 | 2.6 | 4.1 | 6.2 | 4 | 4 | 6.7 | 4 | 6 | 7 |
| 8 | 2.9 | 3.9 | 6.1 | 2.9 | 2.9 | 5.5 | 5.7 | 5.1 | 7 | 7 | 7 | 7 |
| 9 | 3 | 4.1 | 5.3 | 2.4 | 4.1 | 6.6 | 2 | 4 | 6.7 | 4.1 | 4.8 | 5.7 |
| 10 | 4.1 |  |  | 1.1 | 1 |  | 3.1 | 5.9 |  | 5.4 | 5 |  |
| 11 | 2.9 | 4.8 | 5.8 | 2.9 | 5 | 6 | 3.6 | 5.5 | 5.7 | 4.7 | 5.8 | 7 |
| 12 |  | 3.3 | 5.4 |  | 6.2 |  |  | 5.2 | 6.4 | 7 |  | 6.1 |
| 13 |  |  |  | 4.1 |  | 3.2 |  | 7 |  |  |  |  |
| 14 | 1.1 | 2.2 | 7 | 1.7 | 1 | 7 | 2.4 | 2.8 | 7 | 4 | 6.6 | 7 |
| 15 | 2.1 | 3.5 | 5.5 | 1 |  | 2 | 2 | 2 | 6.5 | 2 | 6.6 | 6.8 |
| 16 | 3.1 |  | 6.9 | 1.8 | 2.3 | 3.2 | 2.9 | 2.5 | 5.5 | 5.3 | 4.7 | 5.1 |
| 17 | 4 | 4 | 5.1 | 3.4 | 4 | 4.4 | 4.4 | 4.8 | 5.2 | 4.8 | 6.1 | 5.7 |
| 18 | 2.8 | 4 | 6.1 | 2.3 | 2.1 | 6.5 | 3.3 | 5 | 5.9 | 5 | 5.1 | 6.1 |
| **Mean** | 2.96 | 3.75 | 5.68 | 2.32 | 3.09 | 4.96 | 3.41 | 4.38 | 6.15 | 4.52 | 5.34 | 5.93 |
| **Comparison** | Low vs. Mid | Mid vs. High | Low vs. High | Low vs. Mid | Mid vs. High | Low vs. High | Low vs. Mid | Mid vs. High | Low vs. High | Low vs. Mid | Mid vs. High | Low vs. High |
| **p-value** | 0.000 | 0.000 | 0.000 | 0.043 | 0.000 | 0.000 | 0.027 | 0.000 | 0.000 | 0.016 | 0.008 | 0.001 |

|  |  | **SURPRISED** |  |  | **DISGUSTED** |  |  | **EMBARRASSED** | |  | **PROUD** |  |
| --- | --- | --- | --- | --- | --- | --- | --- | --- | --- | --- | --- | --- |
| **Participant** | **Awed** | **Shocked** | **Astounded** | **Nauseated** | **Appalled** | **Repulsed** | **Ashamed** | **Self-conscious** | **Mortified** | **Satisfied** | **Boastful** | **Victorious** |
| 1 | 4.8 | 5.5 | 5.4 | 3.9 | 5.6 | 5.8 | 3.2 | 4.8 | 4.6 | 2.6 | 5.8 | 5.8 |
| 2 | 5 | 6 | 6 | 5 | 5 | 6 | 4 | 3 | 5 | 3 | 5 | 6 |
| 3 | 6 | 3 | 5.1 | 1.9 | 1.9 | 5 | 3 | 3 | 4 | 4 | 4 | 6.1 |
| 4 | 2.9 | 4.6 |  |  |  |  | 3.9 |  | 5.1 | 3.7 |  | 4.4 |
| 5 | 5.5 | 6.7 | 6.8 | 4.1 | 5.2 | 5.6 | 4.3 | 2.9 | 5.1 | 3.1 | 6.9 | 5.8 |
| 6 | 5.2 | 4.7 | 5.9 | 5.7 | 5.6 | 5.7 | 2.1 | 4.4 | 6.7 | 2.9 | 3.3 | 6.1 |
| 7 | 6.3 | 7 | 6 | 5 | 6.5 | 6.4 | 4.1 | 4 | 5.4 | 3.3 | 6.1 | 6.1 |
| 8 | 4 | 5.9 | 6 | 5 | 5.1 | 4 | 7 | 4 | 7 | 3 | 4.1 | 5.8 |
| 9 | 3.9 | 5.6 | 5.9 | 3.2 | 4.1 | 4.6 | 4 | 7 | 5.1 | 4 | 2.1 | 5.1 |
| 10 | 5.3 | 5.3 |  | 5.3 |  |  |  |  |  |  | 6.2 |  |
| 11 | 5.4 | 6.9 | 6.9 | 5.5 | 6.1 | 6.3 | 1.8 | 5.1 | 6.4 | 5 | 5.5 | 5.6 |
| 12 | 4.1 | 3.9 |  | 7 | 4.1 |  | 4.2 | 7 | 6.2 | 6 | 4 |  |
| 13 | 4 |  | 6.5 | 4 | 6.6 | 6.6 |  |  | 5 |  |  |  |
| 14 | 4 | 5.9 | 7 | 2.4 | 4.4 | 6.5 | 3.2 | 4 | 7 | 2.6 | 4.1 | 7 |
| 15 | 4 | 4.1 | 3 | 2.5 | 5 | 4 | 4 | 6.2 | 4 | 2.1 | 4 | 5 |
| 16 | 3.4 | 6.1 | 5.1 | 2.1 | 3.3 | 3.3 | 3.1 | 2.3 | 4.4 | 4 | 4.2 | 6 |
| 17 | 5.3 | 5.5 | 4.6 | 4 | 4.4 | 5.3 | 4 | 4 | 4.3 | 4 | 4.7 | 5.3 |
| 18 | 4.5 | 5.1 | 6.1 | 2.9 | 4 | 5.1 | 3.1 | 1.8 | 6.9 | 3.4 | 5.7 | 4 |
| **Mean** | 4.64 | 5.40 | 5.75 | 4.09 | 4.81 | 5.35 | 3.69 | 4.23 | 5.42 | 3.54 | 4.73 | 5.61 |
| **Comparison** | Low vs. Mid | Mid vs. High | Low vs. High | Low vs. Mid | Mid vs. High | Low vs. High | Low vs. Mid | Mid vs. High | Low vs. High | Low vs. Mid | Mid vs. High | Low vs. High |
| **p-value** | 0.037 | 0.624 | 0.008 | 0.028 | 0.097 | 0.000 | 0.273 | 0.028 | 0.000 | 0.023 | 0.022 | 0.000 |

Note. *p* values are uncorrected.
