# Supplementary figures and images for: “Grumpy” or “furious”? arousal of emotion labels influences judgments of facial expressions
Source: PLoS One. 2020 Jul 1;15(7):e0235390. doi: 10.1371/journal.pone.0235390 (PMC7329125; doi:10.1371/journal.pone.0235390)

**Appendix B: Example of test trial format**


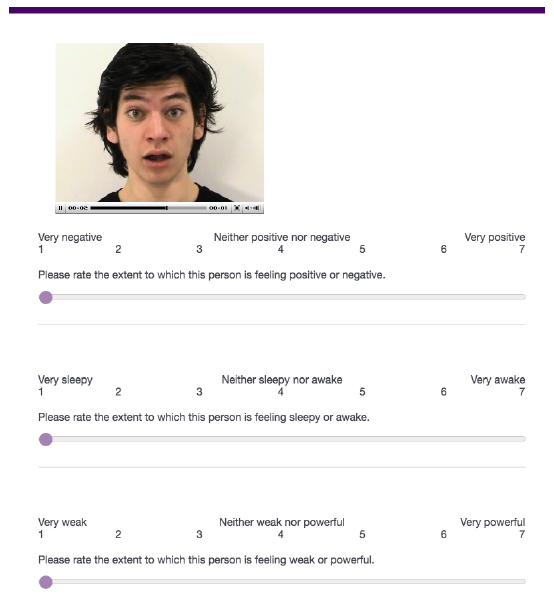

Supplement: S2 Appendix — (DOCX) [file pone.0235390.s002.docx]
