# Supplementary material for: “Grumpy” or “furious”? arousal of emotion labels influences judgments of facial expressions
Source: PLoS One. 2020 Jul 1;15(7):e0235390. doi: 10.1371/journal.pone.0235390 (PMC7329125; doi:10.1371/journal.pone.0235390)
Supplement: S3 Appendix — (DOCX) [file pone.0235390.s003.docx]

**Appendix C:** Means and standard deviations of arousal, valence and dominance ratings for low and high arousal Labels Alone.

| **Emotion** | **Label** | **Label**  **Arousal**  **Level** | **Arousal Ratings**  ***M(SD)*** | **Valence Ratings**  ***M (SD)*** | **Dominance Ratings**  ***M (SD)*** |
| --- | --- | --- | --- | --- | --- |
| Happy | Contented | Low | 3.98 (1.14) | 5.18 (1.06) | 4.55 (0.80) |
|  | Elated | High | 5.33 (1.43) | 5.90 (1.33) | 5.11 (1.29) |
|  |  | *Difference* | 1.35 | 0.72 | 0.56 |
| Sad | Down | Low | 2.91 (0.82) | 2.44 (0.61) | 3.04 (1.03) |
|  | Distraught | High | 4.36 (1.61) | 1.99 (0.87) | 3.80 (1.95) |
|  |  | *Difference* | 1.45 | -0.45 | 0.76 |
| Angry | Grumpy | Low | 3.57 (1.37) | 2.38 (0.78) | 4.09 (1.40) |
|  | Furious | High | 6.13 (0.98) | 1.76 (1.08) | 5.85 (1.28) |
|  |  | *Difference* | 2.56 | -0.62 | 1.76 |
| Scared | Worried | Low | 4.59 (1.28) | 2.64 (0.90) | 3.85 (1.23) |
|  | Terrified | High | 5.70 (1.33) | 1.69 (0.67) | 3.96 (2.20) |
|  |  | *Difference* | 1.11 | -0.95 | 0.11 |
| Disgusted | Nauseated | Low | 3.73 (1.19) | 2.27 (0.75) | 4.19 (1.40) |
|  | Repulsed | High | 4.89 (1.15) | 1.87 (0.81) | 5.12 (1.45) |
|  |  | *Difference* | 1.16 | -0.40 | 0.93 |
| Embarrassed | Ashamed | Low | 4.23 (1.01) | 2.04 (0.74) | 3.99 (1.72) |
|  | Mortified | High | 5.15 (1.23) | 1.97 (0.90) | 4.55 (1.73) |
|  |  | *Difference* | 0.92 | -0.07 | 0.56 |
| Proud | Satisfied | Low | 4.40 (1.31) | 5.49 (1.06) | 4.85 (0.97) |
|  | Victorious | High | 5.86 (1.03) | 6.19 (0.68) | 6.16 (0.95) |
|  |  | *Difference* | 1.46 | 0.70 | 1.31 |
| Surprised | Awed | Low | 4.95 (1.24) | 5.25 (1.30) | 4.46 (1.10) |
|  | Astounded | High | 5.53 (1.24) | 4.43 (1.12) | 4.99 (1.15) |
|  |  | *Difference* | 0.58 | -0.82 | 0.53 |

Note: *Difference* refers to the difference in mean arousal / valence / dominance ratings between high and low arousal label (= *M* High – *M* Low).
