# Supplementary material for: “Grumpy” or “furious”? arousal of emotion labels influences judgments of facial expressions
Source: PLoS One. 2020 Jul 1;15(7):e0235390. doi: 10.1371/journal.pone.0235390 (PMC7329125; doi:10.1371/journal.pone.0235390)
Supplement: S4 Appendix — (DOCX) [file pone.0235390.s004.docx]

**Appendix D.** Means and standard deviations of all label arousal ratings (incl. medium arousal labels)

| **Emotion** | **Label** | **Arousal Level** | ***M*** | ***SD*** |
| --- | --- | --- | --- | --- |
| Happy | Contented | Low | 3.98 | 1.14 |
|  | Pleased | Medium | 4.61 | 1.22 |
|  | Elated | High | 5.33 | 1.43 |
| Sad | Down | Low | 2.91 | 0.82 |
|  | Miserable | Medium | 3.01 | 1.00 |
|  | Distraught | High | 4.36 | 1.61 |
| Angry | Grumpy | Low | 3.57 | 1.37 |
|  | Irritated | Medium | 4.55 | 1.23 |
|  | Furious | High | 6.13 | 0.98 |
| Scared | Worried | Low | 4.59 | 1.28 |
|  | Afraid | Medium | 5.05 | 1.21 |
|  | Terrified | High | 5.70 | 1.33 |
| Disgusted | Nauseated | Low | 3.73 | 1.19 |
|  | Appalled | Medium | 4.97 | 1.17 |
|  | Repulsed | High | 4.89 | 1.15 |
| Embarrassed | Ashamed | Low | 4.23 | 1.01 |
|  | Self-conscious | Medium | 4.33 | 1.00 |
|  | Mortified | High | 5.15 | 1.23 |
| Proud | Satisfied | Low | 4.40 | 1.31 |
|  | Boastful | Medium | 5.03 | 1.09 |
|  | Victorious | High | 5.86 | 1.03 |
| Surprised | Awed | Low | 4.95 | 1.24 |
|  | Shocked | Medium | 5.83 | 1.08 |
|  | Astounded | High | 5.53 | 1.24 |

*Note.* Medium arousal labels were excluded from Main Analyses, but included in analyses below.
