# Supplementary material for: “Grumpy” or “furious”? arousal of emotion labels influences judgments of facial expressions
Source: PLoS One. 2020 Jul 1;15(7):e0235390. doi: 10.1371/journal.pone.0235390 (PMC7329125; doi:10.1371/journal.pone.0235390)
Supplement: S5 Appendix — (DOCX) [file pone.0235390.s005.docx]

**Appendix E: Main analyses including medium arousal labels (STATIC STIMULI)**

We conducted an 8 (emotion) x 3 (label arousal level) repeated-measures ANOVA for each dimension measured (arousal, valence, dominance). For analyses in which assumptions of sphericity were violated, we applied Greenhouse-Geisser corrections.

# Arousal

# As predicted, arousal ratings for faces paired with labels differed between emotion categories and label arousal level. There was a main effect for emotion category, *F*(3.97, 202.29) = 67.99, *p* < .001, ηp2 = .571, and a main effect for label arousal level, *F*(1.54, 78.53) = 48.36, *p* < .001, ηp2 = .487. There was also a significant two-way interaction, which indicated that the effect of label arousal level on perceived arousal of faces varied by emotion category, *F*(8.77, 447.28) = 6.01, *p* < .001, ηp2 = .105. Bonferroni-corrected post-hoc analyses revealed that ratings for sad, angry, scared and proud faces differed significantly depending on the paired label. Sad faces were rated as significantly higher in arousal when paired with the high arousal label ‘distraught’ (*M* = 3.76) than the low arousal label ‘down’ (*M* = 3.33), *p* = .003. Angry faces paired with the high and medium arousal labels ‘furious’ and ‘irritated’ (*M* = 4.82 & *M* = 4.24, respectively) were rated as significantly more aroused than when the low arousal label ‘grumpy’ (*M* = 3.85), *p*s < .025. Likewise, scared faces paired with the high and medium arousal labels ‘terrified’ and ‘afraid’ (*M* = 5.18 & *M* = 5.23, respectively) were rated as higher in arousal than when paired with the low arousal label ‘worried’ (*M* = 4.68), all *p*s < .001. Finally, when paired with the high arousal label ‘victorious’ (*M* = 5.76), proud faces were rated as more aroused than when paired with the low arousal label ‘satisfied’ (*M* = 5.18), *p* < .001.

# Valence

# As predicted, valence ratings for faces paired with labels differed across emotion categories and label arousal level. There was a significant main effect for emotion category, *F*(2.36, 120.18) = 572.10, *p* < .001, ηp2 = .918, and a significant main effect for label arousal level, *F*(2, 102) = 6.67, *p* = .002, ηp2 = .116. The two-way interaction between emotion categories and label arousal was significant, indicating that the effect of label arousal on valence ratings differed depending on the emotion category, *F*(8.05, 410.69) = 10.98, *p* < .001, ηp2 = .177. This interaction was driven by responses to proud and surprised faces. Proud faces paired with the high arousal label ‘victorious’ (*M* = 6.24) were rated as more positive than when paired with the low and medium arousal labels ‘satisfied’ and ‘boastful’ (*M* =5.90 & *M* = 5.74, respectively), *p* = .003 and *p* < .001, respectively. Surprised faces were rated as more positive when paired with the low and high arousal labels ‘awed’ and ‘astounded’ (*M* = 4.39 & *M* = 4.21, respectively) than with the label ‘shocked’ (*M* = 3.64), both *p* < .001.

# Dominance

Dominance ratings for faces paired with labels also differed between emotion categories and label arousal level. There was a main effect for emotion category, *F*(2.99, 152.52) = 189.02, *p* < .001, ηp2 = .788, a main effect for label arousal level, *F*(1.55, 78.94) = 21.37, *p* < .001, ηp2 = .295, and a significant two-way interaction which indicates that the effect of label arousal on dominance ratings differed across emotion categories, *F*(9.12, 465.04) = 9.98, *p* < .001, ηp2 = .164. This interaction was driven by responses to angry, disgusted, and proud faces. Angry faces paired with the high arousal label ‘furious’ (*M* = 4.84) were rated as higher in dominance than when paired with the low arousal label ‘grumpy’ (*M* = 4.05), *p* < .001. Disgusted faces paired with the high and medium arousal labels ‘repulsed’ and ‘appalled’ (*M* = 3.96 and *M* = 3.82, respectively) were rated as more dominant than when presented with the low arousal label ‘nauseated’ (*M* = 3.37), both *p* < .001. When proud faces were paired with the high arousal label ‘victorious’ (*M* = 6.06), they were rated as higher in dominance than when paired with low and medium arousal labels ‘satisfied’ and ‘boastful’ (*M* = 5.49 & *M* = 5.71, respectively), *p* < .001 and *p* < .020, respectively.+

*Figure SM1*. Arousal (1 = *very sleepy,* to 7 = *very awake*), valence (1.00 = *very negative*, to 7.00 = *very positive*) and dominance (1.00 = *very weak*, to 7.00 = *very powerful*) ratings for static faces paired with low, medium and high arousal labels, for each emotion category. Note: * *p* < .05, two-tailed, 95% confidence intervals shown.
