# Supplementary material for: “Grumpy” or “furious”? arousal of emotion labels influences judgments of facial expressions
Source: PLoS One. 2020 Jul 1;15(7):e0235390. doi: 10.1371/journal.pone.0235390 (PMC7329125; doi:10.1371/journal.pone.0235390)
Supplement: S6 Appendix — (DOCX) [file pone.0235390.s006.docx]

**Appendix F: Faces+Labels vs. Faces Alone (STATIC STIMULI)**

To further investigate the effect of labels on perceived arousal of faces, ratings of faces paired with labels (Faces+Labels condition, *N* = 53) were compared with ratings of Faces Alone (*N* = 71). For each of the label arousal levels (high, medium, low) we conducted an 8 (emotion category) x 2 (condition: Faces+Labels vs. Faces Alone) repeated measures ANOVA.

***High arousal labels.*** When ratings of faces in isolation were compared to faces paired with high arousal labels, there was a significant main effect of emotion category*, F*(5.08, 620.26) = 112.31, *p* < .001, ηp2 = .479, and a significant interaction between emotion category and condition, *F*(5.08, 620.26) = 7.67, *p* < .001, ηp2 = .059. The Faces+Labels were rated as higher in arousal than the Faces Alone for proud faces (*p* < .001).

***Medium arousal labels.*** For faces in isolation compared to faces paired with medium arousal labels, there was a significant main effect of emotion category, *F*(5.16, 623.86) = 128.69, *p* < .001, ηp2 = .515, and a significant interaction between emotion category and condition, *F*(5.16, 623.86) = 4.20*, p* = .001, ηp2 = .034. However, for all emotion categories, Faces+Labels were rated as similar in arousal to the Faces Alone (*p*s = 1.00).

***Low arousal labels.*** Finally, for faces alone compared to faces paired with low arousal labels, there was a significant main effect of emotion category, *F*(5.15, 628.08) = 121.50, *p* < .001, ηp2 = .499, and a significant interaction between emotion category and condition, *F*(5.15, 628.08) = 6.78, *p* < .001, ηp2 = .053. However, as with the medium arousal labels, for all emotion categories the Faces+Labels were rated as similar in arousal to the Faces Alone (*p*s > .197).

*Figure SM2*. Arousal ratings (1 = *very sleepy*, to 7 = *very awake*) for Faces+Labels, Faces Alone (Static), and Labels Alone for eight emotion categories. Note: The Faces Alone data (dashed line) represent a single mean per emotion category, presented here as a flat line.
