# Supplementary material for: “Grumpy” or “furious”? arousal of emotion labels influences judgments of facial expressions
Source: PLoS One. 2020 Jul 1;15(7):e0235390. doi: 10.1371/journal.pone.0235390 (PMC7329125; doi:10.1371/journal.pone.0235390)
Supplement: S7 Appendix — (DOCX) [file pone.0235390.s007.docx]

**Appendix G: Main analyses including medium arousal labels (DYNAMIC STIMULI)**

For each dimension measured (arousal, valence, dominance) we conducted an 8 (emotion) x 3 (label arousal level) repeated-measures ANOVAs. For analyses in which assumptions of sphericity were violated, we applied Greenhouse-Geisser corrections. Note: due to a technical error, data for one poser were not collected for the medium arousal label for pride (‘boastful’). Therefore, ratings provided for faces paired with the ‘boastful’ label represent an average of three rather than four faces.

# Arousal

As in Study 1, arousal ratings for faces paired with labels were different across emotion categories and label arousal level. There was a main effect for emotion category, *F*(3.47, 187.12) = 72.95, *p* < .001, ηp2 = .575, and a main effect for label arousal level, *F*(1.67, 89.93) = 35.63, *p* < .001 ηp2 = .398. A significant two-way interaction indicated that the effect of manipulated label arousal level on perceived arousal from faces varied by emotion category, *F*(8.90, 485.72) = 3.67, *p* < .001 ηp2 = .064. Bonferroni-corrected post-hoc analyses revealed a consistent pattern for sad, angry and scared faces, where faces paired with labels higher in arousal were perceived to be significantly more aroused than those paired with lower arousal labels. Sad faces paired with high arousal label ‘distraught’ (*M* = 3.65) were associated with higher arousal ratings than those paired with low arousal label ‘down’ (*M* = 3.24), *p* < .001. Angry faces paired with high arousal label ‘furious’ (*M* = 4.73) were associated with higher arousal ratings than those paired with low arousal label ‘grumpy’ (*M* = 4.18), *p* < .001. Similarly, scared faces paired with high arousal label ‘terrified’ (*M* = 5.59) and medium arousal label ‘afraid’ (*M* = 5.49) were both associated with higher perceived arousal ratings than those paired with low arousal label ‘worried’, *p* < .001 and *p* = .003, respectively.

# Valence

As in Study 1, valence ratings for faces paired with labels were different across emotion categories and label arousal level; there was a significant main effect for emotion category, *F*(1.84, 92.19) = 565.38, *p* < .001, ηp2 = .918, and a significant main effect for label arousal level, *F*(2, 100) = 18.20, *p* < .001, ηp2 = .267. A significant two-way interaction between emotion categories and label arousal indicated that the effect of label arousal on valence ratings differed depending on the emotion category, *F*(9.71, 485.70) = 12.80, *p* < .001, ηp2 = .204. For sad and surprised faces, more highly aroused labels were associated with more negative perceptions, whereas lower arousal labels were seen to be more positive. Sad faces paired with low arousal label ‘down’ were perceived to be feeling more positive (*M* = 2.31) than those paired with the medium or high arousal labels, ‘miserable’ and ‘distraught’ (*M* = 1.74 & *M* = 1.88, respectively), *p*s < .001. Surprised faces paired with the low arousal label ‘awed’ (*M* = 4.56) were seen to be significantly more positive than faces paired with medium label ‘shocked’ (*M* = 3.97, *p* < .001) and high label ‘astounded’ (*M* = 4.29, *p* = .007). To the opposite effect, more highly aroused labels were associated with more positive perceptions for proud faces; faces paired with high arousal label ‘victorious’ (*M* = 6.23) were seen to be significantly more positive than those paired with medium label ‘boastful’ (*M* = 5.98), *p* = .043.

# Dominance

As in Study 1, dominance ratings for faces paired with labels were different across emotion categories and label arousal level; there was a main effect for emotion category, *F*(3.44, 182.18) = 246.99, *p* < .001, ηp2 = .823, and a main effect for label arousal level, *F*(2, 106) = 18.61, *p* < .001, ηp2 = .260. A significant two-way interaction identified an effect of label arousal on perceived dominance of faces, depending on emotion category, *F*(9.78, 518.13) = 7.87, *p* < .001, ηp2 = .129. Specifically, angry, proud and disgusted faces were perceived as more dominant when paired with higher arousal labels than when paired with lower arousal labels. Angry faces paired with high arousal label ‘furious’ (*M* = 4.89) were rated as more dominant than for low arousal ‘grumpy’ (*M* = 4.51) and medium arousal ‘irritated’ pairings (*M* = 4.40), *p*s < .001. Proud faces paired with medium arousal label ‘boastful’ (*M* = 6.18) and high arousal label ‘victorious’ (*M* = 6.19) were perceived as significantly more dominant than those paired with low arousal label ‘satisfied’ (*M* = 5.78), *p*s < .001. Disgusted faces paired with medium arousal label ‘appalled’ (*M* = 4.18) and high arousal label ‘repulsed’ (*M* = 4.14) were perceived to be significantly more dominant than those paired with low arousal label ‘nauseated’ (*M* = 3.71), *p*s < .001. In contrast, the direction of the effect was reversed for scared faces; scared faces paired with medium arousal label ‘afraid’ (*M* = 2.54) were perceived to be feeling significantly less dominant than those paired with low arousal label ‘worried’ (*M* = 2.88), *p* = .020.

*Figure SM3.* Arousal (1 = very sleepy, to 7 = very awake), valence (1.00 = very negative, to 7.00 = very positive) and dominance (1.00 = very weak, to 7.00 = very powerful) ratings for dynamic faces paired with low, medium and high arousal labels, for each emotion category. Note: * p < .05, two-tailed, 95% confidence intervals shown.
