# Supplementary material for: “Grumpy” or “furious”? arousal of emotion labels influences judgments of facial expressions
Source: PLoS One. 2020 Jul 1;15(7):e0235390. doi: 10.1371/journal.pone.0235390 (PMC7329125; doi:10.1371/journal.pone.0235390)
Supplement: S8 Appendix — (DOCX) [file pone.0235390.s008.docx]

**Appendix H: Faces+Labels vs. Faces Alone (DYNAMIC STIMULI)**

To further investigate the effect of labels on perceived arousal of faces, ratings of faces paired with labels (Faces+Labels, *N* = 56) were compared with ratings of Faces alone (Faces Alone, *N* = 48). We ran three separate repeated-measures ANOVAs comparing the responses from participants in the Faces Alone condition to the responses from each of the three label arousal levels in the Faces+Labels condition. This resulted in three (high, medium, low arousal labels) 8 (emotion category) x 2 (condition: Faces+Labels vs. Faces Alone) between-subjects ANOVAs.

***High arousal labels.*** Comparing faces in isolation to faces paired with high arousal labels revealed a significant main effect of emotion category*, F*(4.29, 437.40) = 100.56, *p* < .001, ηp2 = .497, and a significant interaction between emotion category and condition, *F*(4.29, 437.40) = 2.14, *p* < .038, ηp2 = .021. However, for all emotion categories, Faces+Labels were rated as similar in arousal to the Faces Alone, *p*s = 1.00.

***Medium arousal labels.*** For faces in isolation compared to faces paired with medium arousal labels, there was a significant main effect of emotion category, *F*(4.59, 463.51) = 98.83, *p* < .001, ηp2 = .495, and a significant interaction between emotion category and condition, *F*(4.59, 463.51) = 3.81*, p* = .037. However, for all emotion categories, Faces+Labels were rated as similar in arousal to the Faces Alone, *p*s > .68.

***Low arousal labels.*** Finally, for faces in isolation compared to faces paired with low arousal labels, there was a significant main effect of emotion category, *F*(4.45, 453.66) = 98.73, *p* < .001, ηp2 = .492, a significant main effect of condition, *F*(1, 102) = 4.81, *p* = .031, ηp2 = .045, and a significant interaction between emotion category and condition, *F*(4.45) = 5.43, *p* < .001, ηp2 = .051. The interaction showed that Faces+Labels were rated as lower in arousal than the Faces Alone for sad and angry faces (*p*s < .010). Although these effects were only significant for two emotion categories, visual inspection of the means indicates that lower arousal labels reduced perceived arousal of faces across most emotion categories in comparison to Faces Alone (see Figure SM4).

*Figure SM4*. Arousal ratings (1 = *very sleepy*, to 7 = *very awake*) for Faces+Labels, Faces Alone (Dynamic), and Labels Alone for eight emotion categories. Note: The Faces Alone data (dashed line) represent a single mean per emotion category, presented here as a flat line.
