# Supplementary material for: “Grumpy” or “furious”? arousal of emotion labels influences judgments of facial expressions
Source: PLoS One. 2020 Jul 1;15(7):e0235390. doi: 10.1371/journal.pone.0235390 (PMC7329125; doi:10.1371/journal.pone.0235390)
Supplement: S9 Appendix — (DOCX) [file pone.0235390.s009.docx]

**Appendix I: Dynamic vs Static Stimuli**

We predicted that labels would have an overall weaker influence on dynamic stimuli than on static stimuli because dynamic expressions are generally more successfully recognised than their static counterparts (46), and therefore might have more information inherent within them. Thus, we sought to compare the influence of labels on static and dynamic stimuli. To control for differences in ratings of static and dynamic stimuli, we computed difference scores by subtracting the ratings for faces with low arousal labels from faces with high arousal labels for each emotion category. Difference scores were calculated for arousal, valence and dominance Faces+Labels data and these represented the degree of influence of the labels. We conducted an 8 (emotion category) x 2 (stimuli: static vs. dynamic) mixed ANOVA for each dimension (arousal, valence, dominance). Greenhouse-Geisser corrections were applied for analyses in which assumptions of sphericity were violated. Significant interactions were followed up with post hoc paired-samples *t*-tests and Bonferroni-corrected *p* values are reported.

**Arousal**

Differences in arousal ratings were compared across emotion categories and between static and dynamic stimuli (see Figure SM5). There was a significant main effect of stimulus type, such that difference scores were greater for static than dynamic stimuli, *F*(1, 107) = 8.41, *p* = .005, ηp2 = .073. This suggests that labels exerted greater influence over the arousal ratings for static stimuli, as predicted. There was no significant interaction, *F*(5.75, 614.71) = 0.69, *p* = .648, ηp2 = .006, suggesting that the difference between static and dynamic stimuli did not vary between emotion categories.

**
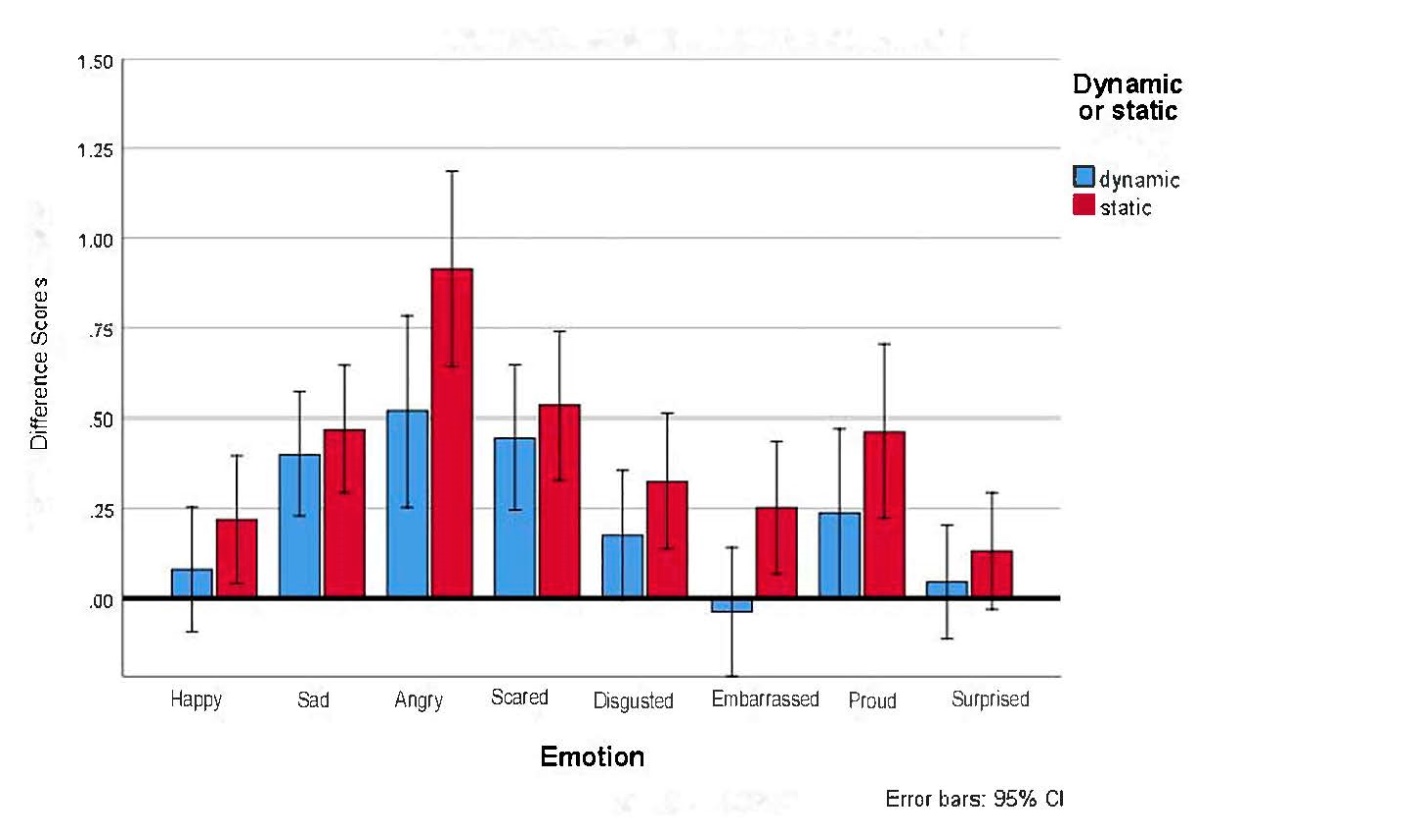
**

*Figure SM5:* Differences in arousal ratings for (= faces with high arousal labels - faces with low arousal labels) for each emotion category, for static and dynamic stimuli.

**Valence**

Differences in valence ratings were compared across emotion category and between static and dynamic stimuli (see Figure SM6). There was no significant main effect of stimulus type, *F*(1, 102) = 2.46, *p* = .120, ηp2 = .024. However, there was a significant interaction, *F*(5.45, 555.71) = 3.82, *p* = .001, ηp2 = .036, which suggests that the difference between static and dynamic stimuli differed across emotion categories. Post hoc comparisons indicated that this interaction was driven by responses to sad faces: difference scores were greater for dynamic than static stimuli, *t*(102) = 4.89, *p* < .001. This indicates that there was a greater difference in participants’ ratings of sad expressions paired with high and low arousal labels when the expressions were dynamic rather than static, as faces paired with high arousal label “distraught” were rated as more negative than those paired with “down”.


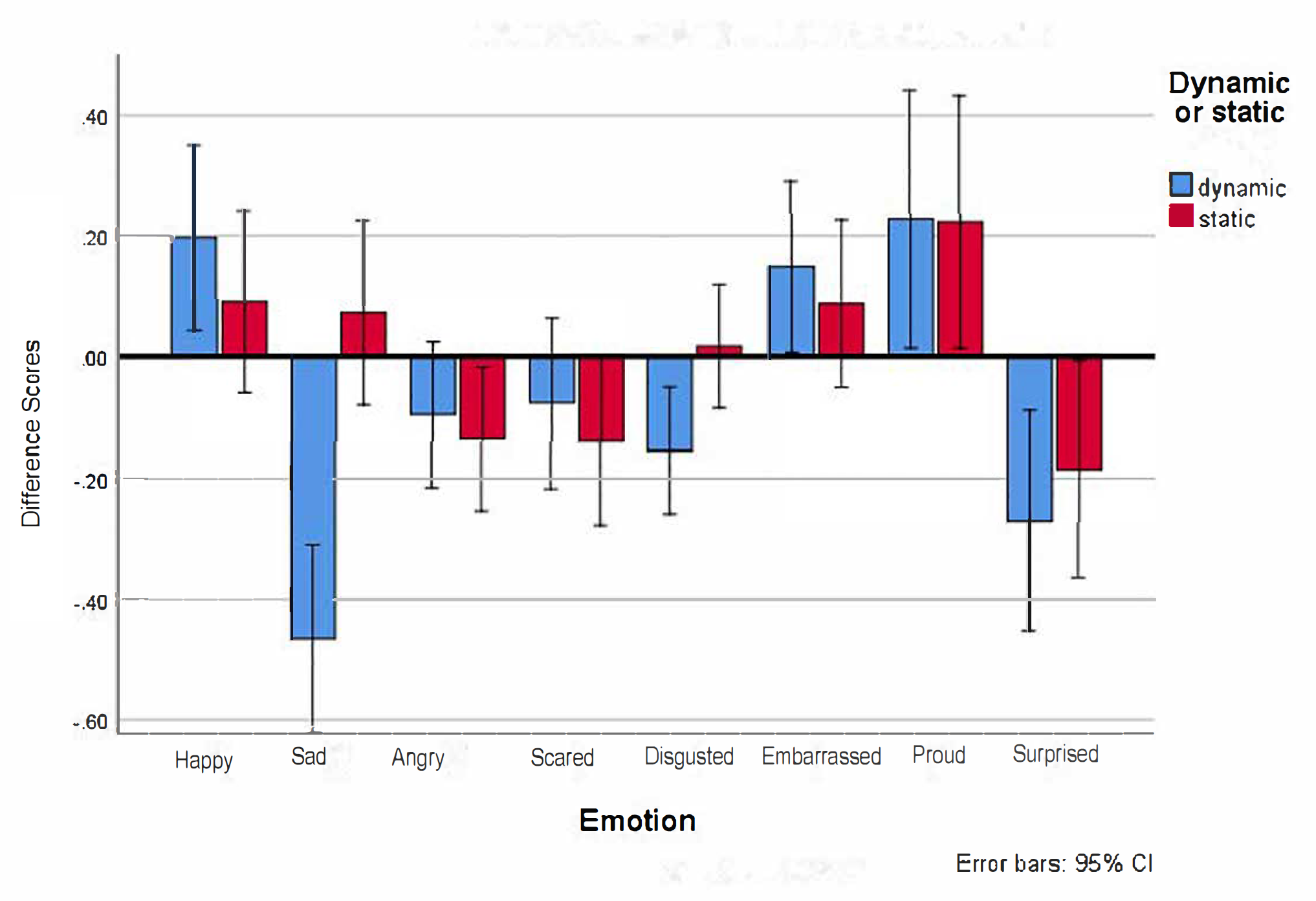


*Figure SM6:* Differences in valence ratings for (= faces with high arousal labels - faces with low arousal labels) for each emotion category, for static and dynamic stimuli.

**Dominance**

Differences in dominance ratings were compared across emotion category and between static and dynamic stimuli (see Figure SM7). There was no significant main effect of stimulus type, *F*(1, 105) = 0.55, *p* = .461, ηp2 = .005, and no significant interaction, *F*(5.97, 626.67) = 1.70, *p* = .118, ηp2 = .016.


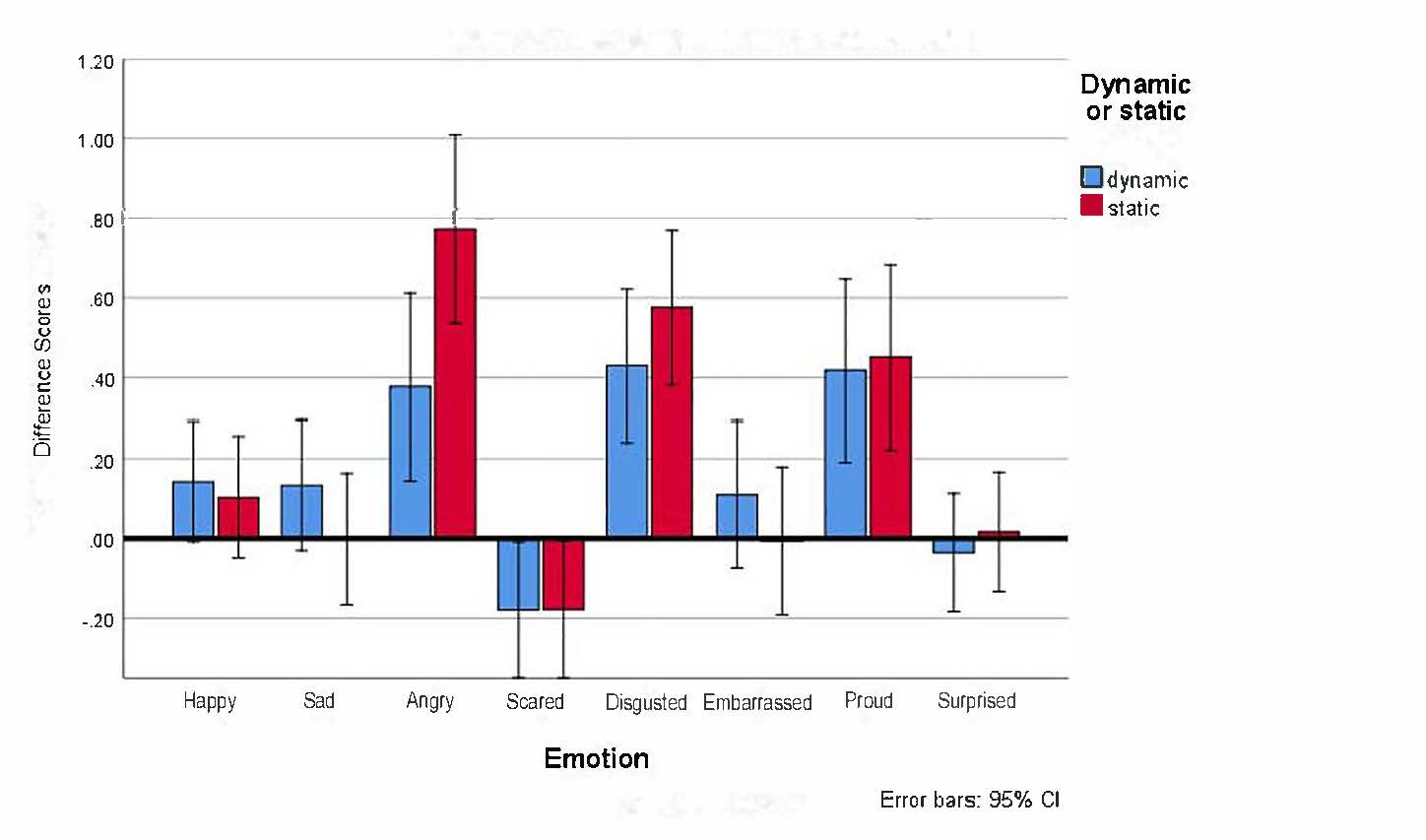


*Figure SM7:* Differences in dominance ratings for (= faces with high arousal labels - faces with low arousal labels) for each emotion category, for static and dynamic stimuli.
