# Supplementary material for: “Grumpy” or “furious”? arousal of emotion labels influences judgments of facial expressions
Source: PLoS One. 2020 Jul 1;15(7):e0235390. doi: 10.1371/journal.pone.0235390 (PMC7329125; doi:10.1371/journal.pone.0235390)
Supplement: S10 Appendix — (DOCX) [file pone.0235390.s010.docx]

**Appendix J: Diamond plots showing all raw data for Faces+Labels ratings**


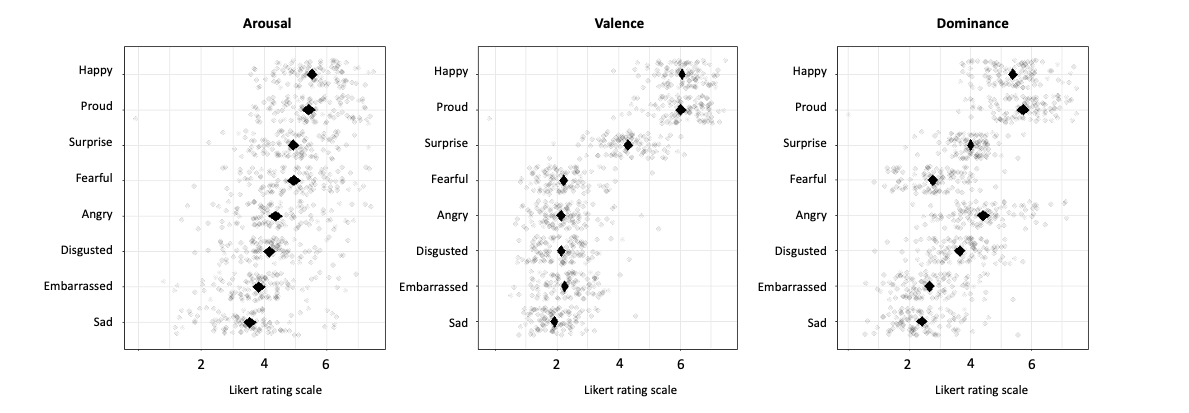
**STATIC STIMULI**

*Figure SM9*. Diamond plots for static stimuli showing means for each emotion across valence, arousal, and dominance dimensions. Length of diamonds represents confidence intervals and dots represent individual data points.

**DYNAMIC STIMULI**


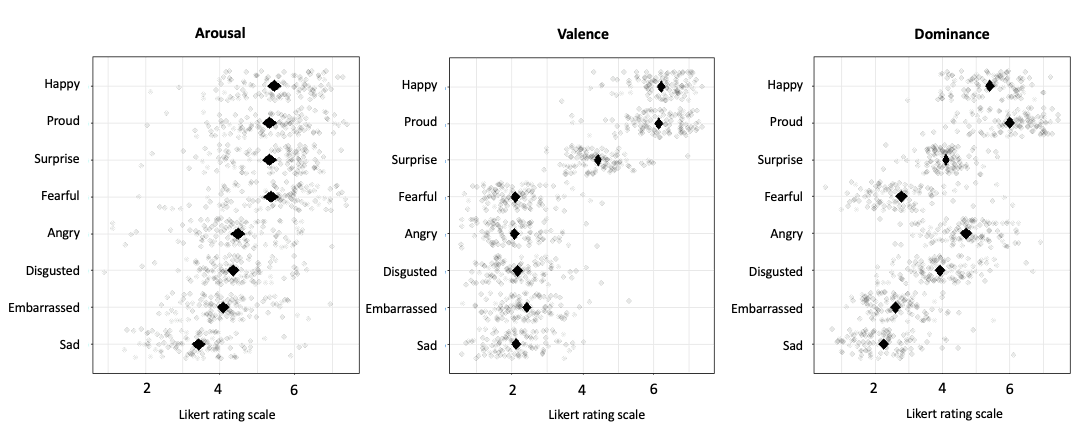


*Figure SM8*. Diamond plots for dynamic stimuli showing means for each emotion across valence, arousal, and dominance dimensions. Length of diamonds represents confidence intervals and dots represent individual data points
