# Supplementary material for: “Grumpy” or “furious”? arousal of emotion labels influences judgments of facial expressions
Source: PLoS One. 2020 Jul 1;15(7):e0235390. doi: 10.1371/journal.pone.0235390 (PMC7329125; doi:10.1371/journal.pone.0235390)
Supplement: Attachment — Submitted filename: Response to Reviewers.docx [file pone.0235390.s012.docx]

Peter A. Bos, Ph.D.

Academic Editor

PLOS ONE

**Re: PONE-D-19-26857R1**

*‘Grumpy’ or ‘Furious’? Arousal of Emotion Labels Influences Judgments of Facial Expressions* (Barker, Bidstrup, Robinson and Nelson)

Thank you for the comments and for inviting us to submit a second revision to *PLOS ONE.* Two of the three reviewers were entirely satisfied with the first revision, but one reviewer had remaining concerns. For convenience, we reproduce Reviewer 1’s comments verbatim with our responses positioned under each comment. Changes made to the manuscript are in italic text.

**Reviewer #1:**

1. This is not a concern, but I would like to mention that the introduction reads much better now and it motivates the two studies nicely.

- Thank you for the positive feedback.

2. I appreciate that the authors have provided a hypothesis regarding their predicted U-shaped curve describing the relationship between arousal, valence and dominance. It might be helpful to clarify exactly how they expect this to be demonstrated in the data from the present two studies. Looking at Figure 1, it seems like some of the results might not fit such a relationship – this should be discussed later on in the text.

- Upon reflection, the U-shaped relationship was not the best way to word our hypothesis. Therefore, we have clarified our hypothesis regarding valence and arousal, and added an example for the reader (p. 8): “*We hypothesised that label arousal level would also influence the valence and dominance ratings of faces consistent with the valence and dominance of the label. For example, we expected that if a high arousal label was low in valence, the valence judgement of the paired facial expression would be lowered.*”

3. I’m not entirely sure the power analysis provided by the authors is correct. A post hoc analysis provides information about the achieved power, not about the sample size needed to achieve that power. Moreover, since they are using a within-subjects design (2 conditions x 8 emotions), there should be only one group of participants.

- We agree with the reviewer that post-hoc power analyses are controversial (e.g., <http://daniellakens.blogspot.com/2014/12/observed-power-and-what-to-do-if-your.html#:~:text=Observed%20power%20(or%20post%2Dhoc,true%20difference%20to%20be%20found.)> and have removed the power analysis from the Supplemental Materials. However, our original justification of sample size (that is, group sizes that are similar to those used in previous research) remains in the manuscript.

4. In their response to point 10 in my previous review (R1), the authors seem to agree with my interpretation about integration and mention that the study is indeed about the integration of face and language cues, however, this isn’t clear from their description in the introduction or anywhere else in the manuscript. In fact, the word integration is not at all mentioned in the text. I would encourage them to elaborate further on this point – I think it will help them provide additional theoretical points of discussion later on in the text.

- As requested by this reviewer, we now make this point about “integration” clearer in the Introduction (p. 6) and the Discussion (p. 24). For example: “*The current studies aim to investigate whether emotion word labels are integrated into judgments of emotion in facial expressions.*”

5. Now that I’ve seen the example trial provided in the Supplementary Materials, I believe that the authors will need to acknowledge the potential carryover effects. What I mean by carryover effects here, is the possibility that participants use the ratings they have provided for one dimension (say valence) to inform their rating on another dimension (say arousal), whereas ideally both ratings should be solely based on the label/face information only.

- Although the pattern of results we found do not suggest carryover effects are driving our results, we now acknowledge this possibility in the discussion on pg. 25, stating “*It is also possible that by rating the three dimensions at the same time, participants’ responses were influenced by their ratings of the other dimensions – future research might consider having participants rate each dimension in separate blocks*”.

6. I understand what the authors’ are trying to say in their response to point 21. I don’t think I have misread the data in the appendix but rather misinterpreted the authors’ explanation. They argue that participants were not influenced by the labels for those emotions but looking at the appendix it seems that when seeing the labels alone participants were clearly influenced by the labels. Therefore, it is not that participants were not influenced by the labels but rather that the labels might not have been taken into consideration when paired with a face image. If this is what the authors meant to say here, I'd encourage them to make it clearer.

- As suggested, the wording of this sentence has been changed to read: “*label information did not appear to be integrated into ratings of happy and embarrassed expressions*”. (p. 24)

7. I’m still quite wary of the lack of differences between the face only and face+label conditions. I take the authors’ point about the difference between a within- and a between-subjects design but there were quite a few participants in both groups. I suspect the effect is weaker and that is why it cannot be detected with a between-subjects design. I think the authors need to be more upfront about this finding – I can see that they have mentioned it in the discussion but that is not enough to draw the reader’s attention to this important point. I’d suggest either elaborating on this point in the discussion (rather than just briefly mentioning it as an idea for future research) or reporting this in the results section (the full analysis can still remain in the Supplementary materials but the main outcome should be mentioned in the main text).

- As requested, these findings are now reported in the Results sections of the main text (p. 14, p. 18).

8. Throughout the analysis sections, the authors mention that the findings are consistent with the valence ratings for Labels Alone. It is not clear what this really means, especially when there are no statistical comparisons between these two conditions provided. Therefore, this seems rather speculative.

- To clarify, we meant that the direction of the effect was consistent with what would be expected based on the labels of ratings alone. For example, surprised face + high arousal “astounded” was rated as more *negative*, while proud face with high arousal “victorious” was rated as more *positive*. This consistent with the valence ratings for Labels Alone. For clarity, we have changed the wording to read: “*These findings are in the same direction as the valence ratings for Labels Alone*” (e.g., p. 19, 20, 22)

9. I would challenge the authors’ conclusions following Study 2 that there is a striking similarity between the results from Study 1 and 2 – they might have to clarify that this is for arousal and dominance only.

- We have opted to remove this sentence from the manuscript (p. 20).

10. Supplementary materials will need to be organised in a more efficient way – I’d suggest assigning each separate section an appendix number/letter and referring to that section in the main text. It’s not clear why appendix A is not included in the supplementary materials.

- As requested, we have reorganised the supplementary materials and assigned each section an Appendix number. We have also moved the table previously in Appendix A to the Supplemental Materials.

11. My most major outstanding issue concerns the discussion. At the moment, it mostly reads like a summary of the results with a few very brief theoretical points being made. This was something mentioned by R3 as well and I think that’s still true for the current version of the manuscript. I have already suggested some interesting theoretical points of discussion both in my first review and here (moreover, some of the results suggest that labels might have a stronger effect on dynamic faces (especially for valence) which contradicts some of the existing literature. This might also be a potential point for discussion). I understand the authors’ point that speculating post hoc might be a poor research practice but simply summarising their results without much reference to the broader theoretical issues and the implications of their findings does not demonstrate the value of their work.

- We have broadened our discussion to highlight some of the theoretical points suggested by the reviewer – particularly the influence of dynamic vs. static expressions. We hope this additional discussion better highlights our findings, without too much post hoc speculation.

12. I also agree with Reviewer 3 that an additional study that allows for a more direct comparison between faces only, labels only and faces+labels would immensely strengthen the manuscript and its overall impact. It is a shame that the authors are unable to collect these data.

- We agree with this reviewer that it is unfortunate we are unable to collect additional data.

Sincerely,

Megan Barker, Ph.D. (on behalf of co-authors)
